# Combining semi-quantitative risk assessment, composite indicator and fuzzy logic for evaluation of hazardous chemical accidents

**DOI:** 10.1038/s41598-020-75583-8

**Published:** 2020-10-29

**Authors:** Huyen Thi Thu Do, Tram Thi Bich Ly, Tho Tien Do

**Affiliations:** 1 Institute for Environment and Resources, Vietnam National University - Ho Chi Minh City, 142 To Hien Thanh Street, Ward 14, District 10, Ho Chi Minh City, Vietnam; 2 Ho Chi Minh City University of Technology and Education, 1 Vo Van Ngan Street, Linh Chieu Ward, Thu Duc District, Ho Chi Minh City, Vietnam

**Keywords:** Environmental impact, Risk factors

## Abstract

In this study, a combination of semi-quantitative risk assessment, composite indicator and fuzzy logic has been developed to identify industrial establishments and processes that represent potential environmental accidents associated with hazardous chemicals. The proposed method takes into consideration the root causes of risk probability of hazardous chemical accidents (HCAs), such as unsafe onsite storing and usage, inadequate operation training, poor safety management and analysis, equipment failure, and factors affected by the potential consequences of the accidents, including human health, water resources, and building and construction. These issues have been aggregated in a system of criteria and sub-criteria, demonstrated by a list of non-overlapping and exhaustive categorical terms. Semi-quantitative risk assessment is then applied to develop a framework for screening industrial establishments that exhibit potential HCAs. Fuzzy set theory with triangular fuzzy number deals with the uncertainty associated with the data input and reduces the influence of subjectivity and vagueness to the final results. The proposed method was tested among 77 industrial establishments located within the industrial zones of Ho Chi Minh City, Vietnam. Eighteen establishments were categorized as high HCA risk, 36 establishments were categorized as medium HCA risk, and 23 ones were of low HCA risk. The results are compatible with the practical chemical safety situation of the establishments and are consistent with expert evaluation.

## Introduction

Hazardous chemical accidents (HCAs) can occur at any location in the production, use, storage, disposal or transportation cycle of the chemical. Toxic chemicals released due to HCAs can result in human injury or death, fire and explosion, destruction of buildings and constructions, and may lead to further consequences, including contamination of land and water bodies and deterioration of the ecological environment. It is important that countries are fully prepared and have emergency plans in place to deal with any accident scenario. In order to prepare for HCAs, hazard sources must be identified and risk prioritized that can help adapt and focus preparedness efforts on the locations of greatest concern. In light of this, a comprehensive understanding of state-of-art and practices in HCA risk assessment is crucial for risk preparedness, emergency planning and relevant decision making.

### Hazardous chemical accidents and the situation in developing countries and in Vietnam

Environmental accidents associated with hazardous chemicals have recently attracted the attention of scholars and government officials. A large number of statistical studies are available that analyze the situations, causes, evolutions, and characteristics of HCAs in different fields and geographical regions worldwide. A study by the Organization for Economic Co-operation and Development^1^ revealed that the release of hazardous substances accounted for a majority of HCAs that occurred within the OECD member countries from 1974–2013. Oggero et al.^2^ analyzed accidents that occurred during the transport of hazardous substances by road and rail from the beginning of the twentieth century to July 2004 and realized an upward trend in the frequency of accidents over time. HCAs are the most common type of accidents, accounting for 78% of the cases worldwide, followed by fires (28%), explosions (14%), and gas clouds (6%). Cunha et al.^3^ studied 119 hazardous and noxious substances spill incidents that occurred in seawater worldwide from 1974 to 2011 and estimated 847,774 tons of 187 chemical substances spilled at sea. Most occurrences involve in flammable liquid substance (21%), toxic substances (16%), oxidizing substances (13%), corrosive substances (12%) and non-dangerous goods (10%). Darbra et al.^4^ studied 225 domino accidents in process/storage plants and during the transportation of hazardous materials. Their analysis showed that the most frequent sequences were explosion followed by fire (27.6%), fire followed by explosion (27.5%), and fire followed by fire (17.8%). Seay et al.^5^ recognized a discrepancy in the trend of accidents occuring in bio-energy sector in USA and Europe during 1997–2014. He concluded that opposite behaviors regarding investment in bio-energy sector lead to the decrease in the trend of accidents in USA and the contemporary increase in the trend of European ones. Dakkoune et al.^6^ showed that the release, leakage, and runaway of hazardous chemicals were the critical scenario for 169 events in French chemical industries between 1974 and 2014. Zhao et al.^7^ identified the trends, chemicals and accident types, causes, and safety management of HCAs in China between 2006 and 2017 and recognized that the primary types of accidents were leakage and explosion of compressed or liquefied gases, and the major reasons for these accidents were operation violation and insufficient production safety training. Whereas another study for the same period (2006–2018)^8^ found that the hazardous material environment and mechanical equipment and organization influence and unsafe supervision are among the main factors causing HCAs in China. Jang et al.^9^ collected data from 2115 cases of chemical accidents that occurred in South Korea over 20 years to establish chemical accident classification codes and a chemical accident reporting system. A more recent study by Jung et al.^10^ showed that over 76% of industrial HCAs in South Korea from January 2008 to June 2018 were resulted from human factors and 72% of them were fire and explosion accidents. Though, a statistical analysis of 85 major accidents in the petrochemical industry recorded in the Major Accident Reporting System (MARS) in the period of 1985–2002 showed that the events leading to an accidental outcome had their origins in the organization and management of the system rather than the technical and human failures^11^. These studies provide a comprehensive understanding of evolution rules of HCAs and support developing accurate and effective predictive preventions and responses.

Vietnam is a typical example of a developing country with an annual economic growth rate greater than 5%, since 2005. Rapid development over the last 15 years has led to many environmental consequences, including HCAs. The occurrences of HCAs have been increasing in recent years, with some being considered as major accidents. On 28 Aug 2019, a fire at a light bulb warehouse in  Ha Noi sparked public concern about the mercury release. On 6 Apr 2016, toxic chemical waste released during cleaning sewage pipe of Formosa steel plant led to massive marine life destruction in central Vietnam. On 18 Sep 2014, a chemical fire at a Japanese printing ink company in Binh Duong province resulted in the release of thousands ton of toxic chemicals to the atmosphere and water. On 17 Apr 2014, a massive fire at a chemical storage in Ho Chi Minh City caused many injuries and a release of 500 tons of toxic chemicals to the nearby river. Although, there are no relevant studies or official documents available in the literature to provide statistical data or reliable information regarding the current situation of HCAs in Vietnam.

On the other hand, studies on HCAs in Vietnam are still limited, fragmented, and lack a holistic approach^12^. The identification of establishments associated with hazardous chemical risk is currently based on a traditional approach that is claimed ineffective in chemical risk management. As stipulated in the Decree No. 26/2011/ND-CP and further updated by the Decree No. 113/2017/ND-CP, the assessment of chemical hazards is relied on the quantity and characteristics of chemicals in use and onsite storage. This approach does not consider the practical constraints of the chemical management as well as the potential consequences of the HCAs, whereas they are the root-causes of HCAs in industry considering the low compliance of the establishments to the chemical safety regulations and the lack of an adequate and effective system of enforcement in Ho Chi Minh City. Thus it sometimes results in the failures of risk management and the implementation of action plan for chemical accident prevention and response, leading to an increasing number of major HCAs in the City. For the reason, a screening method that provides a comprehensive assessment of all underlying factors influencing the HCA risks shall, as required by the City authority, support the identification and statistical analysis of industrial establishments with high risk of HCAs for the purpose of chemical risk management, planning and policy development.

### Risk assessment, process risk assessment and hazard screening for hazardous chemical accidents

Risk Assessment (RA), which was established as a scientific field 30–40 years ago^13^ is a process that evaluates the likelihood and consequence of an adverse outcome due to pressures or changes in environmental conditions resulting from human activities. In RA, risk is a function of two parameters, that is the frequency of occurrence of the undesired event and its consequences^14^. Risk assessment is increasingly a core approach used at all levels of policy and regulation. In industry, the incorporation of risk assessment into the stages of process design and operation is an example of the increasing relevance and value of safety decisions in light of inherent uncertainty arising from unexpected aspects of the development.

Process risk assessment, as one of the essential steps of risk management, involves the identification of potential hazards and analysis of any associated risks within the plant. Process risk assessment is fundamental to the safe design and operation of any process plant or other facilities. The major development of process safety and risk management are related to methods and models or the combination of both, in addition to guidelines, procedures and policies^15^. A large number of publications available in the literature provide comprehensive coverage of generally accepted methods and models for process risk assessment^16–19^. Broadly, the models and methods are categorized into three groups including: qualitative analysis [Failure Modes and Effects Analysis (FMEA)], What-if Checklist, Hazard and Operability Study (HAZOP); semi-quantitative analysis [Layer of Protection Analysis (LOPA), Quantified FMEA, Dow Fire and Explosion Index (F&EI), Dow Chemical Exposure Index (CEI)]; and quantitative analysis (Even tree, Fault tree analysis). FMEA examine every failure mode of all items and elements within a selected part of a system, and determine consequences for each failure mode to support an adequate decision of the response to this failure^17^. HAZOP, as advisory system using rule-based approach, reviews the design of a process to identify and evaluates problems that may represent risks to personnel or equipment^18^. LOPA is a scenario-based approach that assist in judging between alternatives of independent protection layers for risk mitigation. As a simplified risk assessment of a scenario of one cause—one consequence pair, LOPA is more efficient than qualitative assessment in supporting consistent decisions on the adequacy of existing or proposed layers of protection against an accident scenario. However it is less appropriate to analyze complex human behavior or equipment failure to understand the risk of a scenario, in comparison to quantitative assessment^20^. The indicator-based approaches, noteworthy among them are F&EI and CEI, are semi-quantitative risk evaluation methods that have been widely used for hazard identification in process industry. F&EI translates material characteristics and process data to various hazard and exposure factors and calculates the relative ranking of a potential fire and explosion risk associated with the process^21^. CEI relies on factors that can influence the effects of potential chemical releases to identify and rank the relative acute health hazards^22^. These factors comprise of (1) a measure of acute toxicity, (2) the quantity of volatile material available for release, (3) the distance to areas of concern, (4) the molecular weight of the material being evaluated, and (5) various process parameters that can affect the conditions of a release such as temperature, pressure, reactivity, and so forth. Fault tree analysis (FTA) and event tree analysis (ETA) are both logical models that use logic flows to understand the failure of a process or to determine the consequences of a process failure respectively. Extended versions of some techniques including HAZOP, LOPA, amongst others, were discussed thoroughly by Khan^15^. In order to deal with uncertainties, fuzzy logic was extensively applied in process safety and risk assessment. A large number of studies presented the integration of fuzzy logic into HAZOP^23–25^, LOPA^26–28^, FMEA^29–32^, and both FTA and ETA^33–37^. Albeit a long history of development and implementation, these methods and models face some common challenges in their application associated with intensive technical information requirement, considerable expertise and experience requirement, time-consuming preparation and processing. These tools concentrate to assist the industries in making informed decision regarding process risk management and control rather than provide supportive information for the development and implementation of industrial safety policies of the government authorities.

In process risk assessment, chemical hazard is of greatest concern. In order to support the chemical hazard identification, a variety of technical protocols can be found in the open literature that essentially rely on chemical properties and toxicity data to identify whether a chemical has been considered hazardous. The most common approach is indicator-based in which a numerical score is assigned to a chemical substance and/or a process facility to represent the potential hazards associated with the chemical production, storage, handling and disposal. They are generally classified into three main groups: list, framework, and expert system analysis^38^. GreenScreen list translator and its advanced framework version, GreenScreen assessment^39^ identify and classify chemicals of concern by applying scoring systems which use toxicological data or specified hazard lists as the basis for the assessment. GreenWERCS is a list-based screening tool developed by Walmart that generates a “green score” for a product with the chemical data achieved from thirty authoritative lists of chemical hazards^40^. The result is used to recommend a product to Walmart and assists the corporation with chemical compliances. Hazardous Facilities Screening Procedure (HFSP)^41^ evaluates hazardous substances in terms of three major effect groups: fire and explosion, human health, and environment. For each effect group, an Effects Ratio (R) is obtained by dividing the quantity of a substance or group of substances (Q) by the Adjusted Threshold (T). By using the Effects Ratio, it is possible to assess the cumulative potential effects of multiple substances held on the same site and support making decision on the selection of an appropriate site for a proposed hazardous facility. Critical Task Exposure Screening (CTES)^42^ calculates the inhalation risk score by relating the exposure estimate to the corresponding occupational exposure limit or occupational exposure band. EPA’s Risk-Screening Environmental Indicators (RSEI)^43^ is a screening-level model that offers relative comparison of industrial chemical releases from pounds-based, hazard-based, and risk related perspectives. The tool generates an Indicator Element as a combination of facility, chemical, release pathway and exposure pathway that is claimed useful for measuring general changes, setting priorities for further risk analysis, and supporting the strategic planning. Peng^44^ developed a method for evaluating risks and impacts of accidental pollution cases related to water bodies in chemical industrial parks, taking into account three factors: environmental risk sources, magnitude of the impact, and target receptors. It seems obvious that many chemical hazard screening tools are being used in the marketplace to assist the certification of “greener’ chemical ingredients in consumer products or to serve as an analytical basis for risk-related decision support, however they should not be generalized to fit all situations because their evaluations are context‐specific^45^. In addition, the quantity and properties of chemicals are fundamental for most aforementioned hazard screening tools.

A review of the process risk assessment and chemical hazard screening techniques reveals the following facts:

- The application of process risk assessment and hazard screening methods and tools are generally sophisticated and time-consuming to prepare, requiring detailed data that are not always available;
- The results of the assessment processes are indeed context-specific that limited in scope and geographic area;
- The target group of process risk assessment methods and models is likely the industrial companies rather than managing authorities, thus they would be unsuccessful in providing supportive information for the development and implementation of government industrial safety policies and planning;
- Most of the proposed approaches on chemical hazard screening focus on the potential hazards associated with the use and storage of chemicals, and are applied to a specific chemical (single element) or group of chemicals (multiple elements);
- Certain tools and methods estimate and evaluate risks in many criteria but these unlikely to sufficiently cover risk factors inherent in the practical management and operation of the facilities;
- Fuzzy logic has proven its effectiveness in handling the data uncertainty and vagueness commonly faced in process safety and risk assessment.

For these aforementioned reasons, the present study focuses on developing a screening process to identify industrial establishments of high potential environmental risk associated with HCAs. Based on a combination of semi-quantitative risk assessment, composite indicator and fuzzy logic, the proposed approach can be concurrently accomplished for multiple industrial establishments and take into account the inherent risk factors of a practical chemical management situation of establishments. Thus, it is expected to facilitate the policy formulation and management of chemical safety, and chemical incident prevention and response.

## Methods

The method for identifying industrial establishments of potential HCAs is developed from the combination of semi-quantitative risk assessment, composite indicator and fuzzy logic. Theory of semi-quantitative risk assessment is used to identify industrial establishments of high HCA risk. The components of HCA risk including probability of occurrence and severity of the accident impacts are described and determined by using the composite indicator approach. Fuzzy set theory with triangular fuzzy number deals with the uncertainty associated with the data input and reduces the influence of subjectivity and vagueness to the final results. Each method has been widely applied in engineering. However, as a combination, they offer a new and efficient approach in HCA risk evaluation.

### Identification of hazardous chemical accident risk

This method defines the risk of a HCA based on the probability of a hazard occurring and the magnitude of the consequences, as follows:

1 $$R_{i} = P_{i} \times C_{i} ,$$

where, ${\text{R}}_{{\text{i}}}$ is the risk of HCAs of industrial facility i, ${\text{P}}_{{\text{i}}}$ is the probability of occurrence of the HCAs in industrial facility i, ${\text{C}}_{{\text{i}}}$ is the severity of the consequences of the accident.

**Figure 1:**
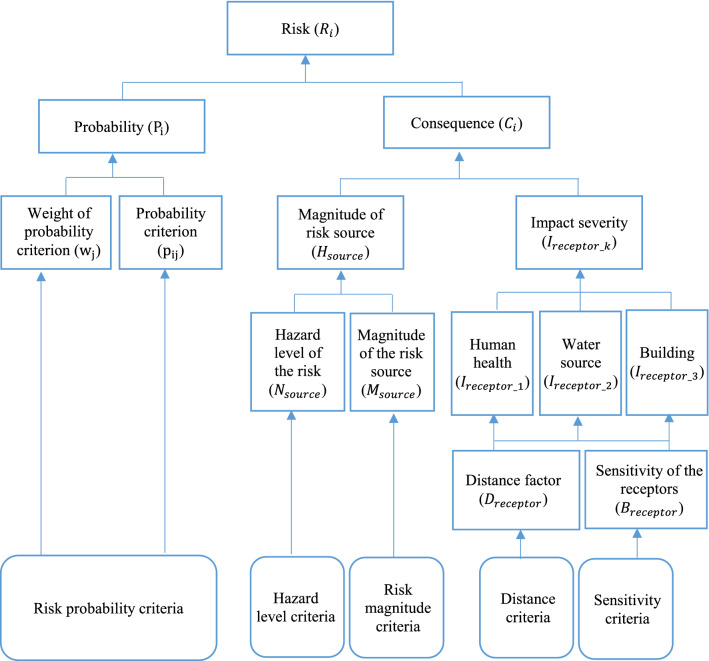
Process for estimation of hazardous chemical accident risk combining semi-quantitative risk assessment and composite indicator.

Unlike a typical semi-quantitative approach of RA that the indexes of probability or the likelihood of occurrence are mostly based on statistical analysis and the consequences of the accidents are based on expert estimates or complex mathematical models, in this study, the probability of occurrence of the HCA (${\text{P}}_{{\text{i}}}$) and magnitude of the consequence (${\text{C}}_{{\text{i}}}$) are achieved through a predefined scoring system based on the composite indicator approach, as presented in Fig. 1.

#### Evaluation of hazardous chemical accident risk probability

The probability of a HCA risk is determined by the current situation of the activities at risk, the compliance with the law and regulations on hazardous chemical management, and the HCA risk prevention and response capability of the establishments. Aforementioned factors are aggregated in a set of 3 criteria and 10 sub-criteria, that are chosen to provide insight into the establishment situations in the context of HCA risk reduction and in line with the regulations (Table 1). Each criterion focuses on different aspects of HCA risk probability being explored and comprehends the same adequacy in the attribute to be measured. The criterion is weighted in favor of Safety on hazardous chemicals use and storage axis by a ratio of 3:4:3, with the score for each sub-criterion carrying equal weight of 10% within their axis. Since chemical safety are multi-faceted and multi-objective, whereas individual opinion vary, equal weighting is certainly an explicit weighting scheme to make the choice of weights less subjective and avoid bias.

**Table 1:** Structure and weighting of the criteria for determining the probability of occurrence ($$w_{j} )$$.

Probability of occurence of HCAs	A. Compliance with the guidelines and regulations on HCAs prevention and response (30%)	A.1 Compliance with the compulsory chemical safety formalities (10%)
		A.2 Compliance with the compulsory hazardous waste management formalities (10%)
		A.3 Development of chemical prevention and response plan (10%)
	B. Safety on hazardous chemicals use and storage (40%)	B.1 Activities inherent risks of HCAs (10%)
		B.2 Chemcial pipelines (10%)
		B.3 Design and operation of the chemical storage(s) (10%)
		B.4 Design and operation of the hazardous waste storage(s) (10%)
	C. Capability of HCAs prevention and response (30%)	C.1 Chemical safety organization struture (10%)
		C.2 Training on chemical safety (10%)
		C.3 Equipment for the HCAs prevention and response (10%)

The performance of the establishments are assessed qualitatively using this set of criteria and then converted to quantitative judgment by a proper scale (Table 4). The probability factor is determined using the following equation:

2 $$P_{i} = \mathop \sum \limits_{j = 1}^{m} w_{j} \times p_{ij} , \forall i = 1,n,$$

where, ${\text{P}}_{{\text{i}}}$ is the factor for probability of HCA occurrence of the establishment i, ${\text{w}}_{{\text{j}}}$ is the weighting of probability sub-criteria j, ${\text{p}}_{{{\text{ij}}}}$ is the quantitative judgment for the performance of establishment i against sub-criteria j.

#### Estimation of magnitude of hazardous chemical accident consequences

**Figure 2:**
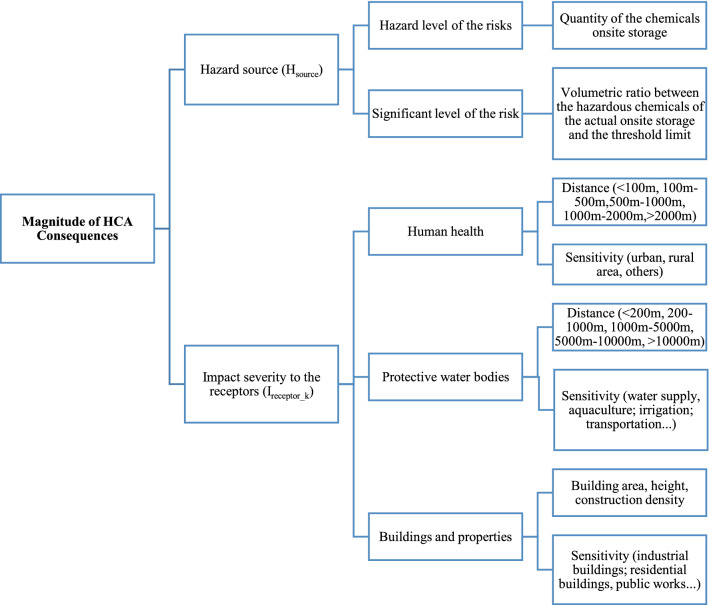
Structure of the factor determining the severity of the HCA consequences ($C_{i}$).

The HCA consequences are evaluated in terms of three major effect groups: human health, protective water bodies, buildings and properties. As presented in Fig. 2, the factor of magnitude of HCA consequences is decomposed into two criteria: the hazard source level and the sensitivity of potential receptors.

Factor for the magnitude of the HCA consequences ($C_{i}$) is a production of the hazard source factor and the total impact severity of various receptors as using the following equation:

3 $$C_{i} = H_{source} \times \sum I_{receptor\_k} ,$$

where, $H_{source}$ is the Hazard source factor, $I_{{{\text{receptor}}\_{\text{k}}}}$ is the Factor on impact severity to the receptor k that takes into account underlying characteristics of the potential receptors (people, natural resources, plants, or animals).

In the HCAs, hazard source factor ($$H_{source} )$$ refers to the source of potential harm, which considers the quantity and hazard of the chemicals being handled or used at the site. The hazard source factor is estimated, as follows:

4 $$H_{source} = N_{chem} \times R_{chem} ,$$

where, $N_{chem}$ is the hazard level of the risks, determined by the quantity of the hazardous chemicals onsite storage as stipulated in Appendix IV an Appendix VII of Decree No. 26/2011/ND-CP, $R_{chem}$ is the significant level of the hazardous chemicals, determined by the volumetric ratio between the maximum hazardous chemicals being stored at the site at a time and the threshold limit for the determination of hazardous chemical as identified in the list of hazardous chemical enclosed to the Decree No. 26/2011/ND-CP.

The factor of severity of impact to receptor k ($I_{receptor\_k}$) considers three types of receptor, that is human health ($I_{receptor\_1}$), protective water bodies ($I_{receptor\_2}$), and buildings and properties ($I_{receptor\_3}$). As presented in Fig. 2, these factors are dependent on the relative distance from the source of the accident to the receptors and the sensitive of the receptors, and being identified as follows:

5 $$I_{receptor\_k} = \sqrt {D_{receptor\_k} \times S_{receptor\_k} } ,$$

where, $D_{receptor\_k}$ is the factor on the distance between the accident source to the receptor k, $S_{receptor\_k}$ is the factor on the sensitivity of the receptor k.

#### Qualitative assessment of HCA risk factors

A qualitative scale of five levels (very high, high, medium, low, very low) is applied for the assessment of risk probability and severity of its potential impacts. In order for this type of labelling to be unambiguous and applicable, two specific guidelines with detailed instructions were developed for the evaluation of factors determining HCA probability and severity of its consequences. Based on a comprehensive analysis of regulatory and technical documents, these guidelines were created that contain descriptive definition of all factors and constituent criteria together with detailed explanation for the determination of the corresponding categorical terms. The guidelines can facilitate the process of data collection and limit the amount of information required for the assessment.

Table 2 below illustrates a part of the guideline for qualitative assessment of HCA probability factor. In evaluating the sub-criterion “Compliance with the compulsory chemical safety formalities”, the study refers to 11 requirements on chemical safety certifications or licenses as stipulated in the legislative decision No. 46/2016/QD-UBND. The assessment of very high, high, medium, low and very low compliance rely on the number of formality requirements being certified as compliant by the establishments. Although all requirements are compulsory, it is occasionally observed during the survey that the establishments, particularly Vietnamese small and medium scale enterprises, fail to comply with several formalities, for instances: certificate of qualification for production of and trading in chemicals on the list of those subject to conditional production and trading; permit for production of and trading in chemicals on the list of those restricted from production and trading; certificate of origin for hazardous chemicals; certificate of approved fire safety design; approval of environmental impact assessment; hazardous material transportation driver permit, and so forth. An inadequate legal awareness of the establishments and a lack of necessary resources to enforce the chemical legislation effectively are amongst the most important causes of the non-compliance. In view of this fact, the performance of establishments against this criterion are categorized into five levels as details in Table 2.

**Table 2:** Example of qualitative assessment for factors defining HCA hazard probability.

Criteria			Categorical terms			
Sub-criteria	Description	Very high	High	Medium	Low	Very low
A.1 Compliance with the compulsory chemical safety formalities	This criterion appraises the proper compliance with the state regulation regarding chemical safety (including 11 requirements as stipulated in Decision No. 46/2016/QD-UBND dated Nov 15th, 2016 of the People’s Committee of Ho Chi Minh City, Vietnam)	Establishments meet from 1 to 2 legislative requirements on chemical safety	Establishments meet from 3 to 4 legislative requirements on chemical safety	Establishments meet from 5 to 7 legislative requirements on chemical safety	Establishments meet from 8 to 9 legislative requirements on chemical safety	Establishments meet from 10 to 11 legislative requirements on chemical safety
B.1 Activities inherent risks of HCAs	This criterion considers the chemical activities/operations involved in HCA hazards and the size of chemical handled and stored on-site at a time as stipulated in Decree No. 26/2011/ND-CP	Establishments having activities/operations in the List of high HCA hazards and being subjected to implementation of Chemical Prevention and Response Plan	Establishments having activities/operations in the List of high HCA hazards and being subjected to implementation of Chemical Prevention and Response Measure	Establishments having no activities/operations in the List of high HCA hazards and being subjected to implementation of Chemical Prevention and Response Plan	Establishments having no activities/operations in the List of high HCA hazards and being subjected to implementation of Chemical Prevention and Response Measure	Other establishments
C.1 Chemical safety organization structure	This criterion considers the status of chemical safety management chemical safety at the facility	No staff of chemical safety or HSE management is appointed in place	To the position of chemical safety and HSE management only one concurrent staff is appointed	To the position of chemical safety and HSE management only one specialized staff is appointed	To the position of chemical safety and HSE management only one specialized staff who received extended chemical safety and other HSE training is appointed	A dedicated department is established that comprises of staffs specialized in and well-trained on chemical safety and HSE management

The sub-criterion “Activities inherent risks of HCAs” otherwise considers the characteristics of the manufacturing processes and activities present the potential HCA hazards. Based on a guideline of Vietnam Environment Administration^46^, a list of 47 industrial activities/operations involved in various HCA hazards have been created. The criterion also considers the types and quantity of hazardous chemicals being handled and stored at a time. In doing so, the study applies two levels of chemical hazards as stipulated in the Appendix IV and Appendix VII of Decree No. 26/2011/ND-CP. Evaluation against this criterion effectively integrates both the establishment’s activities/operations and the levels of chemical hazards as presented in Table 2. It is expected that such integration will support a rigorous evaluation of the inherent risks of HCAs.

In order to evaluate the professional qualification on the chemical safety of the establishments, the study refers to the Chemical Law, Article No. 30, Clause No. 2b. This law stipulates organizations and individuals using chemicals for production must have persons in charge of chemical safety. As shown in Table 2, the establishments are classified into five levels against sub-criterion “Chemical safety organization structure” that are consistent with the observations during the site survey.

Table 3 represents a part of the guideline of HCA consequence evaluation as an illustration for the qualitative assessment against the severity criterion regarding human health. Given that the severity of a HCA impact to human health is dependent on the relative distance from the potential accident source to the receptors and the sensitive of the receptors as identified in Eq. (5), the qualitative assessment against human health sensitivity comprises of five levels attributing to the patterns of population concentration as detailed in Table 3. This classification is in line with the regions of receptors of air pollution sources as stipulated in the National Technical Regulation on Industrial Emission of Inorganic Substances and Dusts QCVN 19:2009/BTNMT. Referring to the guidance of the Ministry of Health in Decision No. 3733/2002/BYT, the safety distance between the pollution sources and the residential areas are, dependent on the industrial sectors and production capacity of the pollution sources, 2000 m, 1000 m, 500 m and 100 m as the minimum. In the guideline, the relative distance from the potential accident source to the receptors include five values corresponding to five levels of the qualitative assessment (very high, high, medium, low and very low), respectively.

**Table 3:** Example of qualitative assessment for factors defining the severity of HCA consequences regarding human health.

Categorical terms	Human health ($I_{receptor\_1}$)	
	Relative distance between the accident source to the receptor $D_{receptor\_1}$	Sensitivity of the receptor $S_{receptor\_1}$
Very high	< 100 m	Special urban and grade 1 urban centre
High	100–500 m	Grade 2, 3, 4 urban centre and suburb of special urban and grade 1 urban
Medium	500–1000 m	Industrial park, grade 1 urban centre, and suburb of grade 2, 3, 4 urbans
Low	1000–2000 m	Rural areas
Very low	> 2000 m	Other areas

#### Incorporating fuzzy logic to the calculation procedure of HCA risk

In order to deal with the uncertainty of qualitative assessment against HCA risk probability and severity factors, the present study applies fuzzy logic to convert qualitative data into quantitative form that can be used as input parameters in the proposed equations. Triangular fuzzy numbers were chosen owning to their ease of application and computation. Various scales have been introduced to convert qualitative assessments to triangular fuzzy numbers^47–49^. The scale presented by Chan et al.^48^ was applied in the study as shown in Table 4.

**Table 4:** Fuzzy scale for the qualitative assessment.

Rating	Score
Very high	(3; 5; 5)
High	(1; 3; 5)
Medium	($\frac{1}{3}$; 1; 1)
Low	($\frac{1}{5}$;${ }\frac{1}{3}{ }$; 1)
Very low	($\frac{1}{5}$;${ }\frac{1}{5}{ }$;${ }\frac{1}{3}$)

The standard interval-based arithmetic of fuzzy numbers as described by Gani^50^ is fundamental for the calculation of the probability factor, consequence magnitude factor and HCA risk as in the Eqs. (1) to (5). The total HCA risk factor ($R_{i}$) resulted from the calculation process are in form of triangular fuzzy numbers, presented as (*i*_1_, *i*_2_, *i*_3_)*,* in which i_1,_ i_2_, i_3_ respectively denote the lower bound value (LBV), most occurring value (MOV) and upper bound value (UBV) of the risk factor. In order to facilitate the risk assessment and decision making, the total HCA risk factor will be defuzzified and converted to script number. The centroid point defuzzification method^51^ was applied as given in the following equations:

6 $$\tilde{x}_{i} = \frac{1}{3}\left( {i_{1} + 2i_{2} + i_{3} } \right),$$

7 $$\tilde{y}_{i} = \frac{{i_{1} + 4i_{2} + i_{3} }}{{3\left( {i_{1} + 2i_{2} + i_{3} } \right)}},$$

8 $$\tilde{R}_{i} = \sqrt {\left( {\tilde{x}_{i} } \right)^{2} + \left( {\tilde{y}_{i} } \right)^{2} } .$$

#### Categorization of establishments for risk of hazardous chemical accidents

The final results of the defuzzification represent the ratings of HCA risks that can offer a screening-level, risk-related perspective for relative comparisons for HCA risks of various industrial establishments. It is worth mentioning that since the $\tilde{R}_{i}$ values do not provide absolute measures of risk, they can only be interpreted as relative measures to be compared with other such values. The classification of establishments regarding HCA risks are defined by $\widetilde{ R}_{i}$, as in Table 5 below.

**Table 5:** Classification of establishments for risk of hazardous chemical accidents.

Range	Description
$$\left[ {\tilde{R}_{\min } , \tilde{R}_{\min } + {\raise0.7ex\hbox{$1$} \!\mathord{\left/ {\vphantom {1 3}}\right.\kern-\nulldelimiterspace} \!\lower0.7ex\hbox{$3$}}(\tilde{R}_{\max } - \tilde{R}_{\min } )} \right]$$	Low risk
$$\left[ {\tilde{R}_{\min } + {\raise0.7ex\hbox{$1$} \!\mathord{\left/ {\vphantom {1 3}}\right.\kern-\nulldelimiterspace} \!\lower0.7ex\hbox{$3$}}(\tilde{R}_{\max } - \tilde{R}_{\min } ), \tilde{R}_{\min } + {\raise0.7ex\hbox{$2$} \!\mathord{\left/ {\vphantom {2 3}}\right.\kern-\nulldelimiterspace} \!\lower0.7ex\hbox{$3$}}(\tilde{R}_{\max } - \tilde{R}_{\min } )} \right]$$	Medium risk
$$\left[ {\tilde{R}_{\min } + {\raise0.7ex\hbox{$2$} \!\mathord{\left/ {\vphantom {2 3}}\right.\kern-\nulldelimiterspace} \!\lower0.7ex\hbox{$3$}}(\tilde{R}_{\max } - \tilde{R}_{\min } ), \tilde{R}_{\max } } \right]$$	High risk

This equal interval classification scheme divides the range of risk values into three equal-sized subranges. This method was chosen owning up to the expected distribution of the data. As verified in the case study, the normal distribution of risk value results can be represented accurately by equal interval classification.

### Hazardous chemical accident risk screening for industrial establishments in Ho Chi Minh City, Vietnam

The proposed approach is applied to a large number of industrial establishments in Ho Chi Minh City, Vietnam to determine the risk of HCAs. Initially, a list of 1123 establishments derived from Ho Chi Minh City Export Processing Zone and Industrial Park Authority (HEPZA) was used in the preliminary screening step to figure out a list of statistically reliable and representative establishments for the survey data collection. In this step, the selection is straightforwardly based on the sectorial category and company size in terms of hazardous chemicals of onsite-storage or hazardous wastes for disposal. Firstly, the list was shortened to 232 establishments targeting to nine sectors including chemical production; pesticide; food processing; metallurgy; electronics; fuel transportation and storage; textile; tanning; and hazardous waste treatment. Then a throughout site survey was conducted by the study team and HEPZA that approached and collected information of 171 establishments, accounting for 15.23% of the total number of industrial establishments located inside the industrial zones (IZ) in Ho Chi Minh City. The remaining 61 establishments were not able to collect information since they refused to receive the survey team nor to provide information or they no longer operate.

During the site survey, data was collected through the questionnaire, site investigation, and other related materials including environmental impact assessment reports, fire prevention and fighting plan, chemical accident prevention and response plan provided by those establishments. Based on the information collected during the site survey, 94 establishments were excluded from the list as their company sizes in terms of hazardous chemicals of onsite-storage was relatively small, lower than that regulated in Appendix IV and Appendix VII of Decree No. 26/2011/ND-CP. Moreover, the HCA risks in them were insignificant because the hazardous chemical usage and storage was unlikely observed during the site survey.

The remaining 77 establishments were selected for the proposed screening process. To support the estimation of HCA risk, a HCA risk database was compiled, which fully covered the criteria of HCA risk probability of occurrence and severity of consequence, including but not limited to data relating to the manufacturing process, that is the usage, storage and disposal of hazardous chemicals and wastes, and the current practices on HCAs prevention and response. Since many of the proposed criteria are qualitative, evaluation of establishments against such criteria are defined regarding their real-life performance and the underlying chemical activity characterization. This is facilitated by the guidelines for evaluating HCA risk probability and impact severity developed by the study group.

The application of the proposed methodology resulted in a list of 18 establishments being categorized as high HCA risk with ${\tilde{\text{R}}}_{{\text{i}}}$ above 2.20. Thirty six establishments were categorized as medium HCA risk with ${\tilde{\text{R}}}_{{\text{i}}}$ ranging between 1.42 and 2.20, and 23 establishments of low HCA risk with ${\tilde{\text{R}}}_{{\text{i}}}$ below 1.42, as shown in Table 6.

**Table 6:** Categorization of establishments for HCA risks in the case study.

Range of relative risk factor ($\widetilde{{R_{i} }}$)	Risk level	Number of establishments
2.20 < $\tilde{R}_{i}$	High risk	18
1.42 < $\tilde{R}_{i}$ ≤ 2.20	Medium risk	36
1.42 ≤ $\tilde{R}_{i}$	Low risk	23

**Figure 3:**
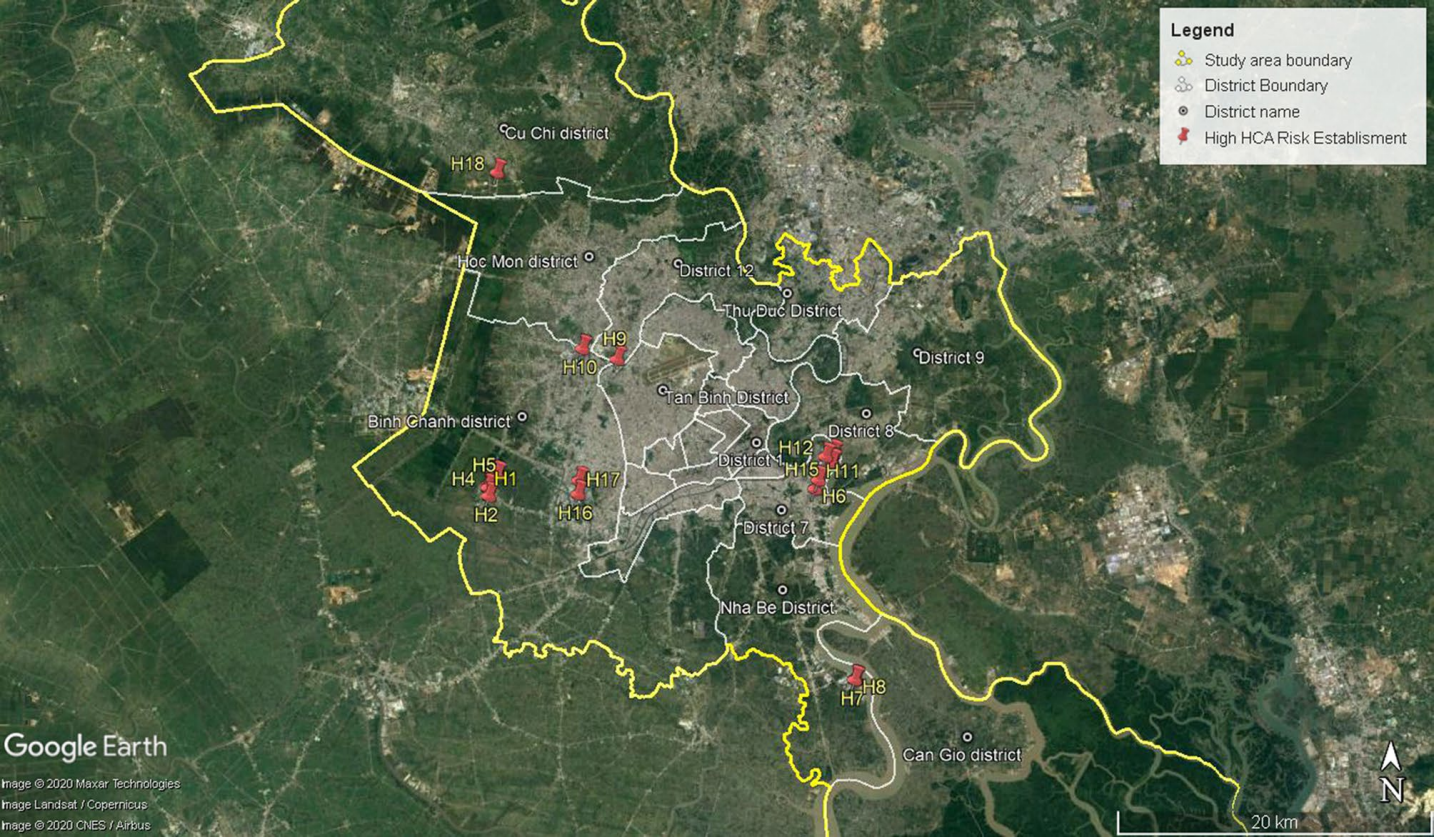
Site map of the industrial establishments presenting high risk of hazardous chemical accidents [processed and extracted from Google Earth Pro 7.3.2.5776 (64-bit), https://www.google.com/earth/download/gep/agree.html (2019)].

Eighteen establishments of high HCA risk are of greatest concerns. The results of HCA risk probability factors, consequence factors, and total risk factors of these establishments are presented in Table 7 and Fig. 3.

**Table 7:** Results HCA risk calculation for high risk group.

No	Anno- tation	Location	Nationality of establishment	Sectorial category	Probability $(P_{i} )$	Consequence ($C_{i}$)	Risk $(R_{i} )$	Risk rating $(\tilde{R}_{i} )$
1	H11	Tan Thuan IZ	Japanese	Alcoholic products	(0.84;2.07;2.80)	(0.4;1.0;1.5)	(0.32;2.13;4.18)	2.97
2	H10	Tan Binh IZ	Vietnamese	Cold storage warehouse	(0.67;2.00;3.00)	(0.4;0.8;1.8)	(0.24;1.59;5.33)	2.95
3	H18	Tan Phu Trung IZ	Vietnamese	Pesticide	(0.91;1.40;1.80)	(0.49;1.32;2.02)	(0.44;1.84;3.64)	2.92
4	H4	Le Minh Xuan IZ	Vietnamese	Pesticide	(0.67;2.00;3.00)	(0.42;1.03;1.40)	(0.28;2.07;4.21)	2.92
5	H17	Tan Tao IZ	Vietnamese	Textile dyeing	(0.97;2.05;2.07)	(0.42;1.14;1.64)	(0.41;2.33;3.39)	2.87
6	H16	Tan Tao IZ	Vietnamese	Textile dyeing	(0.77;1.87;2.13)	(0.43;1.22;1.64)	(0.34;2.27;3.50)	2.84
7	H1	Le Minh Xuan IZ	Vietnamese	Pesticide	(0.67;2.00;3.00)	(0.41;0.99;1.32)	(0.27;1.99;3.95)	2.78
8	H3	Le Minh Xuan IZ	Vietnamese	Pesticide	(0.65;1.93;3.00)	(0.41;0.99;1.32)	(0.27;1.92;3.95)	2.73
9	H12	Tan Thuan IZ	Japanese	Fruits and vegetable processing and cold storage warehouse	(0.77;1.87;2.60)	(0.4;1.0;1.5)	(0.30;1.92;3.88)	2.72
10	H6	Hiep Phuoc IZ	Vietnamese	Textile dyeing	(0.93;2.40;3.40)	(0.36;0.71;1.19)	(0.33;1.72;4.05)	2.65
11	H2	Le Minh Xuan IZ	Vietnamese	Pesticide	(0.65;1.93;2.60)	(0.41;1.03;1.32)	(0.27;2.00;3.43)	2.61
12	H8	Hiep Phuoc IZ	Vietnamese	Tanning	(0.64;1.84;2.87)	(0.36;0.81;1.57)	(0.23;1.49;4.50)	2.61
13	H9	Hiep Phuoc IZ	Vietnamese	Tanning	(0.63;1.77;2.87)	(0.36;0.81;1.57)	(0.22;1.44;4.50)	2.57
14	H7	Hiep Phuoc IZ	Vietnamese	Pesticide and fertilizer	(0.61;1.29;1.60)	(0.52;1.41;2.24)	(0.32;1.82;3.58)	2.56
15	H5	Le Minh Xuan IZ	Vietnamese	Pesticide	(0.59;1.73;2.60)	(0.41;0.991.32)	(0.24;1.72;3.43)	2.42
16	H14	Tan Thuan IZ	Thailand	Cosmetic production	(0.57;1.65;2.33)	(0.39;1.03;1.49)	(0.22;1.70;3.48)	2.42
17	H15	Tan Thuan IZ	Thailand	Cosmetic production	(0.57;1.65;2.33)	(0.39;1.03;1.49)	(0.22;1.70;3.48)	2.42
18	H13	Tan Thuan IZ	Japanese	Footwear	(0.51;1.47;2.00)	(0.5;1.2;1.5)	(0.26;1.71;2.99)	2.28

From the results obtained, it is recognized that:

- Eighteen establishments are categorized as high HCA risk, accounting for 23.4 percent of the number of establishments under consideration;
- Vietnamese enterprises account for a majority of the high HCA risk establishments (correspondent to 83.3 percent);
- Tanning, pesticide and fertilizer production, food production and cold storage are sectors of highest concerns;
- These establishments are mostly located in the proximity of the city centre, particularly those located in the high sensitive areas such as Tan Thuan industrial zone adjacent to Phu My Hung, an upper-class urban area (H6, H11, H13–H15); and Le Minh Xuan industrial zone (H1–H5), an old-fashioned and chemical-intensive industrial area of the City;
- They share common characteristics of high HCA risk probability due to their poor chemical management practices, which are consistent with the observation during the site survey, such as improper storage, handling and disposal of chemicals and hazardous wastes; low compliance with chemical safety and hazardous waste management regulations; inadequate training on emergency response, fire prevention and safety management; inefficient occupational health and safety management system; lack of safety procedures; and insufficient preventive maintenance. This is particularly noteworthy for the small-scale enterprises operating in the tanning and pesticide industries;
- The magnitude of HCA consequences appears to be dependent on the hazard sources rather than the severity of HCA impacts, as most establishments are located close to populated areas and water bodies. The factors of impact severity are almost invariable for establishments in the same industrial zone. However, for those using and storing hazardous chemicals in large quantities, particularly foreign investment enterprises, HCA consequence factors are particularly higher than others.
- Several enterprises encountered HCAs during their operation, for instances a NH_3_ release accident at H10 in 2017, a fire and hazardous chemical release at H3 in 2014.

The proposed method, in comparison to other index-based hazard screening approaches, would be more generalized and integrated in view of the underlying concepts of criteria. It offers the inclusion of diverse HCA hazards in one risk value while the most well-established approaches, F&EI and CEI, fire and explosion hazards and toxic chemical hazards are assessed separately by two distinct tools. Relatively little information may be required that make the proposed method being less analytically demanding but still estimates magnitude and probability of effects with reasonable confidence.

### Ethical approval

This study was appraised and approved four times by the specific scientific committees of Vietnam National University of Ho Chi Minh City (VNU-HCM) and Ho Chi Minh City Department of Sciences and Technology (HCMC-DOST) during 2016 to 2018, in which technical and (part of) ethical concerns of the study was considered. Since there is not any regulations/requirements regarding ethical approval for such study in Vietnam, all procedures performed in studies involving human participants were in accordance with the ethical standards of the institutional research committee and with the 1964 Helsinki declaration and its later amendments or comparable ethical standards.

### Informed consent

Informed consent was obtained from all individual participants included in the study.

## Conclusion and findings

In this study, a screening method has been developed to support the identification of industrial establishments of high potential HCAs. The method is proposed as a new and improved approach for chemical risk assessment as it successfully combines three different methods: semi-quantitative risk assessment, composite indicator and fuzzy logic. Such a combination allows a simple and efficient risk analysis and facilitates handling data uncertainty and inconsistency, which usually challenge traditional risk analysis. It is also novel in the mathematical models that make use of complex qualitative indicators to determine the risk probability and consequence parameters. Although it comprehensively considers all risk factors including root causes of risk probability and potential receptors of the accident consequences. Facing challenges of sophisticated application and detailed data requirement of the process risk assessment and chemical hazard screening, the proposed method is expected to be comprehensive, yet simple enough for users without a technical background.

The proposed method was tested among 77 industrial enterprises located within the industrial zones of Ho Chi Minh City, Vietnam. It was found that eighteen establishments were categorized as high HCA risk with ${\text{R}}_{{\text{i}}}$ ranging above 2.20. Thirty-six establishments were categorized as medium HCA risk with ${\text{R}}_{{\text{i}}}$ ranging between 1.42 and 2.20, and twenty-three ones were of low HCA risk with ${\text{R}}_{{\text{i}}}$ below 1.42. The results of the implementation of the proposed method suggest that this approach is beneficial for both industrial practitioners and the relevant authorities. It will enable establishments to recognize their HCA hazards, help to raise their awareness on chemical safety and thus promote a safer production, proactive chemical management and emergency preparedness. The outcomes of the case study also contribute to a better understanding and efficient management of industrial HCA risks of the relevant government authorities. A number of immediate measures have been introduced for a short-term plan, for examples developing agency coordination policies to promote efficient management of HCA risks, creating an appropriate chemical safety inspection program to ensure compliance; reinforcing the authority’s enforcement capability against chemical safety infringements, targeting to establishments of high risk group, and so forth. It is expected that the application of the proposed method to a larger scale will provide implication for industrial land-use planning and environmental policy oriented to sustainable development of Ho Chi Minh City in the long term.

The testing results also illustrated the capability of a large-scale application to realize potential HCAs, however some limitations should be noted. Although semi-quantitative approach facilitates the implementation and allows flexibility of the risk assessment process, qualitative assessment is predominant in the judgment of the risk factors that could be claimed for low credibility and researcher’s personal biases. Still it is worth mentioning that the criteria for determining HCA risk probability and severity factors are developed on the basis of legal and technical specifications which are more or less quantitative. In addition, the probability estimation is merely a weight sum approach with fixed weight calculation. This approach is considered straightforward but sufficient for the determination of HCA risk probability to the extent that the chemical management system is currently ineffective and lack of attention across the local authority. From this perspective, a possible extension of the study can be foreseen, aimed at improving the qualitative assessment and the weight sum approach for probability calculation.

Last but not least, practical challenges faced during the site survey can affect the results of the case study. Sixty-one establishments, accounting for up to 26.3 percent of the total number of establishments initially selected, were not available for the data collection due to a number of reasons, including their concerns of the sensitive information disclosure, temporary production downtimes, and unapproachable condition of the establishments. Information provided by establishments were, on the other hand, sometimes insufficient and inconsistent to the real-life performance that demanded significant time and effort for data processing. An integration of sensitivity and uncertainty analysis into the proposed method can be another potential direction of further studies in order to improve the reliability of the assessment results. However, it is notable that the final screening results obtained are contemporarily compatible with the practical chemical safety situation of the establishments and consistent to the expert evaluation.

## Data Availability

The datasets generated during and/or analysed during the current study are not publicly available due to confidentiality agreement with research participants but are available from the corresponding author on reasonable request.

## References

[1] OECD. *25 Years of Chemical Accident Prevention at OECD-History and Outlook*. (Organisation for Economic Co-operation and Development, Paris, 2013).

[2] Oggero A, Darbra R, Munoz M, Planas E, Casal J (2006). A survey of accidents occurring during the transport of hazardous substances by road and rail. J. Hazard. Mater..

[3] Cunha I, Moreira S, Santos MM (2015). Review on hazardous and noxious substances (HNS) involved in marine spill incidents—An online database. J. Hazard. Mater..

[4] Darbra RM, Palacios A, Casal J (2010). Domino effect in chemical accidents: Main features and accident sequences. J. Hazard. Mater..

[5] Seay J, Lunghi E, Rehman A, Fabiano B (2017). Analysis of accident data for the bioenergy sector based on second generation feedstocks. Chem. Eng. Trans..

[6] Dakkoune A, Vernières-Hassimi L, Leveneur S, Lefebvre D, Estel L (2018). Risk analysis of French chemical industry. Saf. Sci..

[7] Zhao L (2018). An analysis of hazardous chemical accidents in China between 2006 and 2017. Sustainability.

[8] Li X, Liu T, Liu Y (2019). Cause analysis of unsafe behaviors in hazardous chemical accidents: Combined with HFACs and Bayesian network. Int. J. Environ. Res. Public. Health.

[9] Jang N (2009). Development of chemical accident classification codes and tool for management in process industries. J. Chem. Eng. Jpn..

[10] Jung S, Woo J, Kang C (2020). Analysis of severe industrial accidents caused by hazardous chemicals in South Korea from January 2008 to June 2018. Saf. Sci..

[11] Nivolianitou Z, Konstandinidou M, Michalis C (2006). Statistical analysis of major accidents in petrochemical industry notified to the major accident reporting system (MARS). J. Hazard. Mater..

[12] Huyen DTT, Tram LTB (2019). Development of a procedure for evaluating the impacts of the accidental emission of hazardous chemicals, case study in Ho Chi Minh City, Vietnam. Environ. Manag..

[13] Aven T (2016). Risk assessment and risk management: Review of recent advances on their foundation. Eur. J. Oper. Res..

[14] Gould, J. H. In *Handbook of Environmental Risk Assessment and Management* (ed. Calow, P. P.) 91–108 (Wiley, Hoboken, 2009).

[15] Khan F, Rathnayaka S, Ahmed S (2015). Methods and models in process safety and risk management: Past, present and future. Process Saf. Environ. Prot..

[16] Bahr NJ (2017). System Safety Engineering and Risk Assessment: A Practical Approach.

[17] Crawley F, Tyler B (2003). Hazard Identification Methods.

[18] Greenberg HR, Cramer JJ (1991). Risk Assessment and Risk Management for the Chemical Process Industry.

[19] Ostrom LT, Wilhelmsen CA (2019). Risk Assessment: Tools, Techniques, and Their Applications.

[20] Willey RJ (2014). Layer of protection analysis. Proc. Eng..

[21] Dow Chemical Company (1994). Dow’s Fire & Explosion Index Hazard Classification Guide: A AIChE Technical Manual.

[22] Dow Chemical Company (1994). Dow’s Chemical Exposure Index Guide: A AIChE Technical Manual.

[23] Ahn J, Chang D (2016). Fuzzy-based HAZOP study for process industry. J. Hazard. Mater..

[24] Cheraghi M, Eslami Baladeh A, Khakzad N (2019). A fuzzy multi-attribute HAZOP technique (FMA-HAZOP): Application to gas wellhead facilities. Saf. Sci..

[25] Fuentes-Bargues JL, González-Gaya C, González-Cruz MC, Cabrelles-Ramírez V (2016). Risk assessment of a compound feed process based on HAZOP analysis and linguistic terms. J. Loss Prev. Process Ind..

[26] Khalil M, Abdou MA, Mansour MS, Farag HA, Ossman ME (2012). A cascaded fuzzy-LOPA risk assessment model applied in natural gas industry. J. Loss Prev. Process Ind..

[27] Markowski AS, Mannan MS (2009). Fuzzy logic for piping risk assessment (pfLOPA). J. Loss Prev. Process Ind..

[28] Hong Y, Pasman HJ, Sachdeva S, Markowski AS, Mannan MS (2016). A fuzzy logic and probabilistic hybrid approach to quantify the uncertainty in layer of protection analysis. J. Loss Prev. Process Ind..

[29] Petrovskiy EA, Buryukin FA, Bukhtiyarov VV, Savich IV, Gagina MV (2015). The FMEA-risk analysis of oil and gas process facilities with hazard assessment based on fuzzy logic. Mod. Appl. Sci..

[30] Dağsuyu C, Göçmen E, Narlı M, Kokangül A (2016). Classical and fuzzy FMEA risk analysis in a sterilization unit. Comput. Ind. Eng..

[31] Adar E, İnce M, Karatop B, Bilgili MS (2017). The risk analysis by failure mode and effect analysis (FMEA) and fuzzy-FMEA of supercritical water gasification system used in the sewage sludge treatment. J. Environ. Chem. Eng..

[32] Wessiani NA, Sarwoko SO (2015). Risk analysis of poultry feed production using fuzzy FMEA. Proc. Manuf..

[33] Renjith VR, Madhu G, Nayagam VLG, Bhasi AB (2010). Two-dimensional fuzzy fault tree analysis for chlorine release from a chlor-alkali industry using expert elicitation. J. Hazard. Mater..

[34] Wang D, Zhang P, Chen L (2013). Fuzzy fault tree analysis for fire and explosion of crude oil tanks. J. Loss Prev. Process Ind..

[35] Lavasani SM, Zendegani A, Celik M (2015). An extension to Fuzzy Fault Tree Analysis (FFTA) application in petrochemical process industry. Process Saf. Environ. Prot..

[36] Yazdi M, Zarei E (2018). Uncertainty handling in the safety risk analysis: An integrated approach based on fuzzy fault tree analysis. J. Fail. Anal. Prev..

[37] Ramzali N, Lavasani MRM, Ghodousi J (2015). Safety barriers analysis of offshore drilling system by employing Fuzzy Event Tree Analysis. Saf. Sci..

[38] Gauthier AM (2015). Chemical assessment state of the science: Evaluation of 32 decision-support tools used to screen and prioritize chemicals: Chemical Assessment State of the Science. Integr. Environ. Assess. Manag..

[39] Heine, L., Rossi, M., Hunsicker, A. & Franjevic, S. *GreenScreen for Safer Chemicals Hazard Assessment Guidance*. (Clean Production Action, 2016).

[40] Geiser K (2015). Chemicals Without Harm: Policies for a Sustainable World.

[41] New Zealand Ministry for the Environment, Environmental Risk Management Authority, Hazardous Facilities Screening Procedure Review Group & Environmental Risk Management Authority. *Land use planning guide for hazardous facilities: a resource for local authorities and hazardous facility operators : A report*. (New Zealand Ministry for the Environment, Environmental Risk Management Authority, 2002).

[42] Tjoe-Nij E, Rochin C, Berne N, Sassi A, Leplay A (2018). Chemical risk assessment screening tool of a global chemical company. Saf. Health Work.

[43] USEPA—Office of Information Analysis and Access. *EPA’s Risk-Screening Environmental Indicators (RSEI) Methodology*. (US Environmental Protection Agency, 2015).

[44] Peng J, Song Y, Yuan P, Xiao S, Han L (2013). An novel identification method of the environmental risk sources for surface water pollution accidents in chemical industrial parks. J. Environ. Sci..

[45] Panko J (2016). A comparative evaluation of five hazard screening tools: Comparative Evaluation of Screening Tools. Integr. Environ. Assess. Manag..

[46] Vietnam Environment Agency. *Technical Guidance for Risk Assessment of Hazardous Chemical Release in Industrial Sectors*. (Vietnam Environment Administration, Vietnam, 2014).

[47] Naghadehi MZ, Mikaeil R, Ataei M (2009). The application of fuzzy analytic hierarchy process (FAHP) approach to selection of optimum underground mining method for Jajarm Bauxite Mine, Iran. Expert Syst. Appl..

[48] Chan FTS, Chan HK, Chan MH, Humphreys PK (2006). An integrated fuzzy approach for the selection of manufacturing technologies. Int. J. Adv. Manuf. Technol..

[49] Amelia L, Wahab DA, Hassan A (2009). Modelling of palm oil production using fuzzy expert system. Expert Syst. Appl..

[50] Gani N (2012). A new operation on triangular fuzzy number for solving fuzzy linear programming problem. Appl. Math. Sci..

[51] Cheng CH (1998). A new approach for ranking fuzzy numbers by distance method. Fuzzy Sets Syst..

